# Two-Dimensional Nanomaterials for Polymer-Based Packaging Applications: A Colloidal Perspective

**DOI:** 10.3390/nano15050359

**Published:** 2025-02-26

**Authors:** Dongpo Huang, Luyan Shen, Haifeng Yu

**Affiliations:** 1 Key Laboratory of Polymer Chemistry and Physics of Ministry of Education, School of Material Science and Engineering, Peking University, Beijing 100871, China; 2 Engineering Research Center for Nanophotonics and Advanced Instrument, School of Physics and Electronic Science, East China Normal University, Shanghai 200241, China

**Keywords:** 2D nanomaterials, colloidal interactions, composite films, barrier properties, packaging

## Abstract

The integration of two-dimensional (2D) nanomaterials into polymer-based packaging presents a promising avenue for sustainable, high-performance materials. This perspective explores the roles of colloidal interactions in the assembly of 2D materials into thin films for packaging applications. We begin by analyzing the types of colloidal forces present in 2D nanomaterials and their impact on dispersion and stability. We then explore how these colloidal forces can be modulated through chemical structure, ionic intercalation, and shear forces, influencing the stacking behavior and orientation of 2D materials within the films. The incorporation of these 2D materials into polymer-based packaging systems is also considered, with a focus on how surface functionalization and dispersion techniques enhance their interaction with the polymer matrix to improve barrier properties against gases and moisture, increase mechanical strength, and impart antimicrobial effects. This work underscores the critical role of colloidal interactions in optimizing the design and performance of 2D-nanomaterial-based packaging for sustainable development.

## 1. Introduction

Sustainable packaging materials that minimize environmental impacts while maintaining performance and functionality are gaining increasing attention [1,2,3,4]. Key trends in this field include the increasing use of renewable materials, such as paper, bio-plastics, and plant-based polymers, as well as the optimization of lightweight and reusable packaging designs [5,6,7,8]. In particular, the development of high-barrier packaging materials is crucial in preserving the quality and shelf life of packaged goods in the food industry, without relying on non-biodegradable synthetic films [9,10,11].

Two-dimensional nanomaterials have garnered significant attention in recent years due to their exceptional properties and potential for a wide range of applications, including in packaging [12]. These materials, characterized by their atomic-scale thickness and large surface area, exhibit unique mechanical and barrier properties that can greatly enhance the performance of conventional materials. For example, 2D nanomaterials can act as physical barriers that prevent the diffusion of oxygen, moisture, and other contaminants through the substrates [13,14,15]. Due to their high aspect ratios, even minimal amounts of 2D nanomaterials can create tortuous paths that impede the transmission of gases and liquids [16,17,18]. Additionally, 2D nanomaterials can improve the mechanical integrity of packaging, making it more durable and resistant to wear and tear [16,19]. Some of the most well-known 2D nanomaterials include graphene [20,21,22,23,24], boron nitride (BN) [25,26,27,28], and MXenes [29,30,31,32], and they have demonstrated advantages for improving packaging materials. Typically, graphene’s excellent impermeability to gases and high mechanical strength have made it a promising candidate for barrier applications [33,34,35].

In packaging applications, 2D materials are often integrated into a polymer matrix through wet processing. In such cases, colloidal forces, such as van der Waals interactions, electrostatic repulsion, and steric effects, play a crucial role in the dispersion and stability of 2D materials, and would directly influence their aggregation behavior and overall performance in packaging. For example, good dispersibility ensures the uniform distribution of 2D materials, leading to the formation of a dense barrier layer that significantly enhances the material’s ability to resist the diffusion of gases and moisture. Colloidal stability is equally important, as it prevents the 2D materials from aggregating or precipitating during the manufacturing process, thus ensuring the performance of the packaging material. On the other hand, the colloidal interactions of 2D material dispersions also affect their viscosity, which plays a crucial role in achieving uniform coating and high-quality packaging films. The viscosity must be carefully controlled, as too high or too low viscosity can lead to uneven coatings or performance instability. In addition, the interaction between 2D materials and the polymeric substrate material is also critical. Strong interfacial bonding, facilitated by colloidal forces, enhances the overall mechanical properties of the material, ensuring it can effectively withstand external stresses and pressures during use. By understanding and optimizing colloidal interactions, it is possible to enhance the performance of 2D-material-based packaging, potentially driving the development of more sustainable packaging solutions.

Although several publications have summarized the applications of 2D materials in packaging or coating [15,36], they primarily focus on material fabrication and performance optimization. This perspective offers a fresh approach by examining 2D materials in packaging through the lens of colloid science, highlighting the interplay between colloidal forces, material assembly, and packaging performance. It fills a gap in the current literature by providing mechanistic insights into how colloidal interactions can be leveraged to optimize material design and enhance packaging functionality. In order to identify the significant advancements in sustainable packaging and 2D nanomaterials, we surveyed the investigated literature from 1977 to 2024 in Web of Science. The search engines were used to find relevant documents by using keywords such as “2D nanomaterials”, “colloidal interactions”, “composite films”, “barrier properties”, and “packaging”. Additional documents were identified by examining references listed in key articles. The initial number of documents obtained by searching the databases was 92,154. Based on title and abstract information, we included only the articles that were considered relevant for 2D nanomaterials for polymer-based packaging. In total, 91 articles were included in this systematic review.

## 2. Colloidal Forces Among 2D Materials

**Figure 1:**
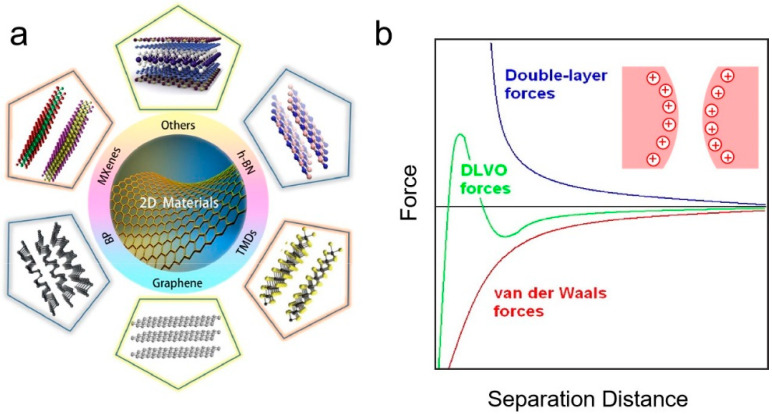
(**a**) A schematic of 2D materials including graphene, black phosphorus (BP), MXenes, hexagonal boron nitride (h-BN), and transition metal dichalcogenides (TMDs). (**b**) A graph representing the attractive (red) and repulsive (blue) forces between two particles with a mass and a charge, which results in the total force between the particles (green) [37,38].

To explore the potential of 2D materials in packaging, it is essential to examine the colloidal forces that govern their assembly and processing behaviors. Colloidal forces, such as van der Waals interactions, electrostatic forces, and steric repulsion, play a significant role in determining the stability, dispersion, and self-assembly of 2D materials in solution environments. These interactions not only influence the integration of 2D materials into packaging systems but also affect their performance characteristics, including mechanical strength and barrier properties. In this section, we will examine the specific colloidal interactions of different 2D materials (Figure 1a), focusing on how these forces shape their dispersion and stability in solution.

The Derjaguin–Landau–Verwey–Overbeek (DLVO) theory is an important theory that describes the interaction between colloidal particles, primarily used to describe the interaction forces between particles in colloidal systems [39]. It explains the stability of colloidal dispersions by balancing two main types of interactions (Figure 1b): attractive van der Waals forces and repulsive electrostatic forces. Van der Waals forces bring particles together, promoting aggregation, while electrostatic repulsion arises from charged surfaces forming an electric double layer, preventing particle contact. The total interaction potential energy, expressed as the sum of these two forces, determines colloidal stability; a sufficient energy barrier between particles indicates that they remain dispersed, while an imbalance can lead to aggregation. Apart from these two interactions, many colloidal systems also experience additional interactions, such as solvation forces, hydrophobic forces, and steric forces, which are not encompassed by the classical DLVO model but can significantly influence colloidal behavior. Typically, various colloidal forces engage in a complex interplay within a single system, competing for dominance. The relative importance of each force is contingent upon a multitude of factors, including the physicochemical properties of the colloidal particles, such as functional groups, crystal structure, rigidity, and surface morphology, as well as the characteristics of the dispersing medium. In the following, we explore the analysis of various 2D-nanomaterial-based colloids in light of these considerations [40,41].

**Figure 2:**
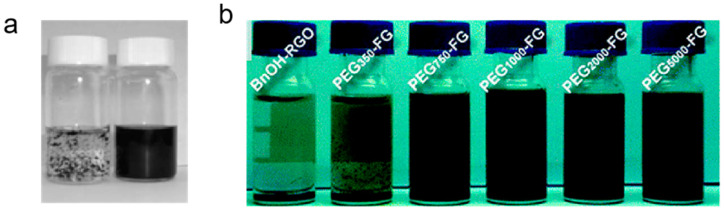
(**a**) Picture showing water dispersions (1 mg mL^−1^) of reduced graphite oxide without PSS (**left**) and with PSS (**right**) [21]. (**b**) Digital photos of PEG-functionalized reduced graphene oxide with different chain lengths dispersed in CHCl_3_ through bath ultra-sonication [22,23].

Graphene exhibits strong van der Waals forces due to π–π stacking interactions between its planar carbon sheets. These interactions can lead to aggregation in colloidal dispersions. To counteract this tendency, researchers often achieve steric stabilization by the incorporation of polymeric stabilizers or surfactants or surface functionalization through chemical grafting, which create a physical barrier that prevents aggregation [42,43]. For instance, the absorption of polymeric surfactants such as polystyrene sulfonate (PSS) can create a repulsive layer around graphene (Figure 2a), preventing aggregation and allowing for more uniform film formation [44]. Polyethylene glycol (PEG) has been grafted to the surface of graphene nanosheets to improve their dispersion in solution (Figure 2b) [45,46].

**Figure 3:**
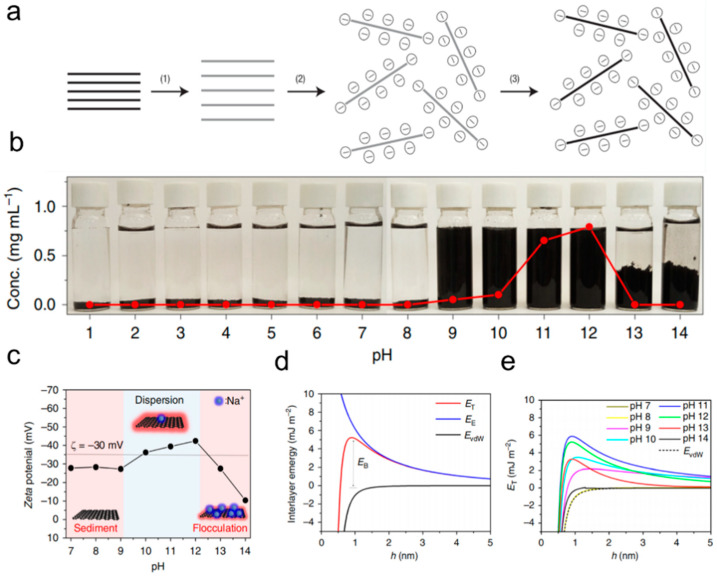
(**a**) Scheme showing the chemical route of the synthesis of aqueous graphene dispersions [47]. (**b**) The stability of aqueous solutions of graphene with respect to pH. Graphene aqueous solutions in the pH range of 1~14 and the corresponding maximum graphene concentrations (dotted line). (**c**) Zeta potential reveals the different dispersion stabilities of graphene solutions as a function of pH. (**d**) Interlayer interaction energies (*E_T_*) versus *h* at pH = 12 from the DLVO theory. (**e**) *E_T_* versus *h* curves at different pH values in the range of 7~14 [41].

However, the addition of foreign dispersants could limit the potential applications of graphene. Li et al. reported that stable aqueous dispersions of chemically converted graphene sheets can be easily formed by electrostatic stabilization [47]. They found that when the pH of aqueous graphene dispersion was adjusted to 9~10 to ionize the oxygen functional groups, a negatively charged layer forms, promoting uniform dispersion (Figure 3a). Dong et al. employed a similar strategy to prepare a high-concentration graphene slurry [41]. At a low pH, graphene tends to aggregate, while at a neutral pH of 11~12, the negative charges on the surface of graphene nanosheets can enhance stability (Figure 3b). The theoretical calculations based on the DLVO theory quantitatively explain the critical role of electrostatic repulsion, attributing the negative charge to the stabilization of graphene dispersion (Figure 3c–e).

**Figure 4:**
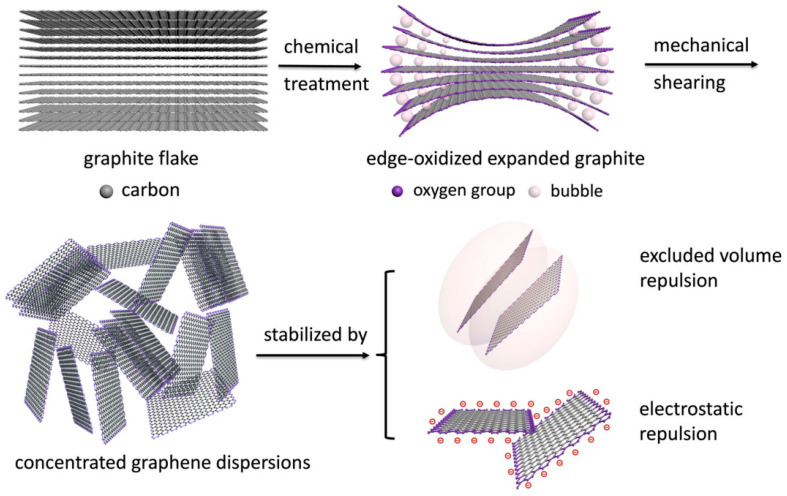
Illustrated diagram of the exfoliation and dispersion of high-concentration few-layer graphene. In the resulting concentrated graphene dispersions, graphene tactoids are surrounded by the exfoliated nanosheets, forming a disordered jammed network structure, where the entropic excluded volume repulsion (marked by the ellipsoid region) couples with the enthalpic electrostatic repulsion to co-stabilize the graphene dispersions [48].

**Figure 5:**
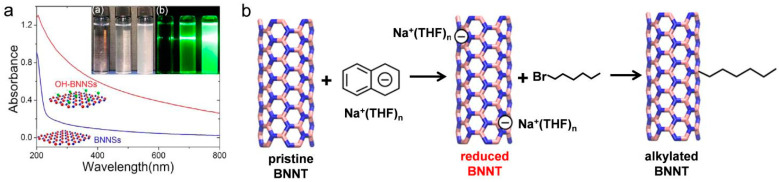
(**a**) UV-Vis spectra of pristine BNNSs and OH-BNNSs in water, with the inset showing photographs of water, and pristine BNNSs and OH-BNNSs in water [53]. (**b**) Schematic illustration of the covalent alkylation of reduced BN nanotubes using 1-bromohexane [54].

The colloidal behavior of nanoparticles is closely associated with their morphology. Recently, Xiong et al. demonstrated an excluded volume effect inherent to 2D morphology, which, when combined with electrostatic repulsion, enables the synthesis of high-concentration aqueous graphene dispersions [48]. As shown in Figure 4, they developed a stepwise method involving edge oxidation, bubble expansion, and mechanical shearing to exfoliate graphite flakes, facilitating the pilot-scale production of graphene dispersions with a mass concentration of 100 mg mL^−1^. Their findings indicated that the 2D shape of graphene nanosheets promotes repulsive excluded volume interactions between the sheets, forming a jammed network of nanosheets and tactoids that prevents aggregation.

h-BN possesses structural characteristics akin to graphene, leading to comparable van der Waals interactions. Its propensity to agglomerate can hinder its performance in practical applications. Ma et al. observed that the boron nitride nanosheet (BNNS) solution began to aggregate after just 12 h of dispersion at room temperature and became fully aggregated within three days [49]. Similar to graphene, the stabilization of BNNS colloids can be improved by employing surface modifications and utilizing dispersants that enhance solvation forces [50,51]. By forming a solvation layer around BNNSs, these modifications help to mitigate the strong van der Waals forces, promoting improved dispersion and stability. For example, introducing hydroxyl (-OH) groups on the BNNS surface effectively increases the electrostatic repulsion between the layers, facilitating stable liquid-phase dispersion. Wu et al. [52] and Ciofani et al. [53] identified a straightforward and effective strategy for introducing -OH groups to the surface of BNNNs (Figure 5a). The introduced -OH groups can effectively enhance the dispersion of BNNSs in solution. An analysis of the UV-vis spectra of -OH-modified BNNSs (OH-BNNSs) compared to pristine BNNSs indicated that the solubility of OH-BNNSs in water is improved due to the presence of surface hydroxyl groups. In addition to functional group surface modification, covalent modifications can also significantly increase the repulsive forces between BN nanotubes. Shin et al. reported the covalent alkylation of reduced BN nanotubes (i.e., negatively charged) using 1-bromohexane, as illustrated in Figure 5b [54].

MXenes, a family of 2D transition metal carbides and nitrides, display unique colloidal behavior attributed to their layered structure and tunable surface chemistry. With a surface rich in functional groups (-OH, -O, -F, etc.), MXenes can be effectively dispersed in water and various polar organic solvents, enhancing their processability [55]. The interactions between MXene layers can be influenced by electrostatic forces, especially when ions or organic molecules are inserted between the layers. This tunability enables the adjustment of interlayer spacing and barrier properties. Additionally, the presence of surface functional groups contributes to steric repulsion, further enhancing the stability of MXene colloids [56,57]. Researchers have shown that by manipulating the surface chemistry, MXenes can achieve optimal dispersion and enhanced performance in applications such as filtration and energy storage [58,59].

Molybdenum disulfide (MoS_2_), a prominent transition metal dichalcogenide, exhibits similar colloidal forces to those seen in graphene and h-BN. The van der Waals interactions between MoS_2_ layers can lead to aggregation, which poses challenges for achieving uniform dispersions. To enhance the colloidal stability of MoS_2_, researchers often employ surfactants or polymeric stabilizers that provide steric hindrance [60]. Electrostatic stabilization is also significant, as the surface charges on MoS_2_ can be manipulated through pH adjustments to maintain a stable dispersion. Recent advances in functionalizing MoS_2_ with hydrophilic groups have further improved its compatibility with various solvents, facilitating the formation of stable colloidal solutions.

## 3. How Colloidal Interactions Influence the Assembly Structure of Film

With an understanding of the key colloidal forces that govern 2D materials, it is important to explore how these interactions directly influence the formation and structure of films. The assembly of 2D materials into ordered structures, such as thin films, is crucial for their application in packaging, as it determines their mechanical and barrier properties. Colloidal interactions are central to controlling the dispersion, alignment, and stacking behavior of the 2D nanomaterials, ultimately dictating the performance of the films. In this section, we explore how colloidal forces can be modulated through the chemical structure of 2D nanosheets, the intercalation of ions, and the application of external fields to influence the final structure of the films.

### 3.1. Impact of Chemical Structure of 2D Materials on Colloidal Interactions and Film Structure

The colloidal properties of 2D materials are heavily influenced by their chemical and structural characteristics [47]. For example, the reduction process of graphene oxide (GO) significantly influences its colloidal force properties. During the reduction reaction, oxygen-containing functional groups on the surface of GO are selectively removed, resulting in substantial changes to the material’s surface chemistry. This evolution of the chemical structure leads to a marked enhancement of the hydrophobicity of reduced graphene oxide (rGO), which typically triggers hydrophobic interactions between the nanosheets. Consequently, this causes the aggregation of colloidal particles and reduces the stability of the dispersed system. It is important to note that variations in the mechanisms of different reducing agents can lead to significant differences in the types and concentrations of residual functional groups on the rGO surface. These differences not only alter colloidal forces, such as van der Waals forces and π-π interactions between the nanosheets, but also affect the solvent evacuation effect, ultimately resulting in considerable differences in the microstructure and macroscopic properties of the self-assembled films. R.R. Nair et al. found that hydroiodic acid, due to its smaller molecular size, can more easily penetrate the interlayers of graphene oxide, and its reducing effect is significantly greater than that of ascorbic acid [34]. The rGO films prepared with hydroiodic acid exhibit a layer spacing close to the intrinsic structure of graphite (~0.36 nm). This dense stacking structure effectively blocks the interlayer penetration channels of small-sized substances, such as helium atoms and water molecules, giving hydroiodic acid-reduced films a significant advantage in gas barrier performance.

### 3.2. Role of Ionic Intercalation in Colloidal Force Regulation and Film Assembly

Ionic intercalation plays a critical role in modulating the colloidal forces between 2D nanosheets and can be used to precisely control the assembly of films. Chen et al. utilized cations such as K^+^, Na^+^, Ca^2+^, Li^+^, and Mg^2+^ to adjust the interlayer spacing of graphene oxide films through cation-π interactions between the cations and graphene flakes [61]. Cao et al. explored the impact of colloidal interactions and electrolyte concentration on the structural evolution of rGO membranes during reduction. By varying the NaCl concentration, it was found that higher electrolyte levels significantly affect the stacking structure of rGO sheets, decreasing interlayer distances and facilitating the formation of larger graphitic clusters. The results showed that an increased electrolyte concentration leads to denser stacking and fewer voids in the membranes, enhancing their stability and swelling behavior in water. However, after high-temperature treatment (90 °C), the influence of electrolyte concentration on the porous structure diminished, indicating that thermal stresses prevailed. The findings suggest that the stacking behavior of rGO nanosheets results from a dynamic interplay between electrolyte-mediated interactions and thermal disturbances, highlighting the importance of the processing sequence for tailoring rGO structures at the sub-nanometer scale [62].

**Figure 6:**
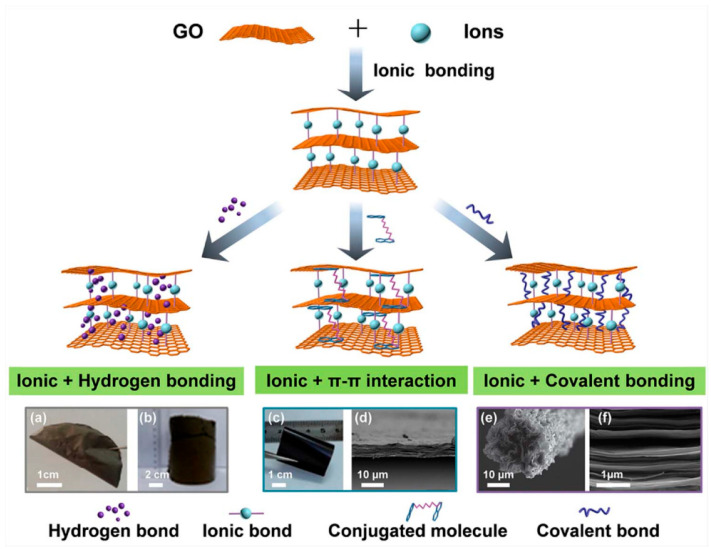
Schematic diagram of graphene-based nanocomposites with ionic interfacial interactions: the following are the digital photographs or morphologies with different synergistic effects [30]. (**a**) The digital photograph of 2D bioinspired graphene-based nanocomposites filmsand (**b**) the digital photograph of 3D excellent compressible graphene-based aerogels with the synergistic toughening effect of ionic bonding and hydrogen bonding; (**c**) The digital photograph and (**d**) cross-section morphology of flexible Eu^3+^-crosslinked GO/PAAP nanocomposites with the synergy of ionic bonding and π-π interaction, as well as hydrogen bonding. (**e**) The digital photograph of 1D ultra-strong graphene-based fibers and (**f**) cross-section morphology of robust 2D rGO-Zn^2+^-PCDO films with the synergistic interaction of ionic bonding and covalent bonding.

The functional surface groups of GO nanosheets enable the creation of interfaces with other polymers or cross-linking agents, thereby improving interfacial strength. These interface designs can typically be classified into hydrogen bonding, ionic bonding, π-π interactions, and covalent bonding. Hydrogen bonding is particularly prevalent in graphene-based nanocomposites. For example, bioinspired GO-polyvinyl alcohol nanocomposites demonstrate an exceptional Young’s modulus and exceptional tensile strength. This is attributed to the robust hydrogen bonding networks established between adjacent GO nanosheets and polyvinyl alcohol chains, as well as between the GO nanosheets and water molecules [63]. In metal ion-modified graphene-based nanocomposites, ionic bonding occurs through the coordination of multivalent metal ions with the functional groups present on the GO nanosheets [64]. Furthermore, π-π interactions occur between conjugated molecules, such as pyrene derivatives, and GO nanosheets, greatly improving both electrical conductivity and interfacial strength [65]. Furthermore, covalent bonding, characterized by high bond energies, provides strong interfacial strength, which can be tailored by adjusting the cross-linking density and the length of the molecular chain. By combining multiple interfacial interactions, a series of high-performance graphene nanocomposites can be prepared (Figure 6).

### 3.3. External Fields Influencing Colloidal Assembly in 2D Material Films

**Figure 7:**
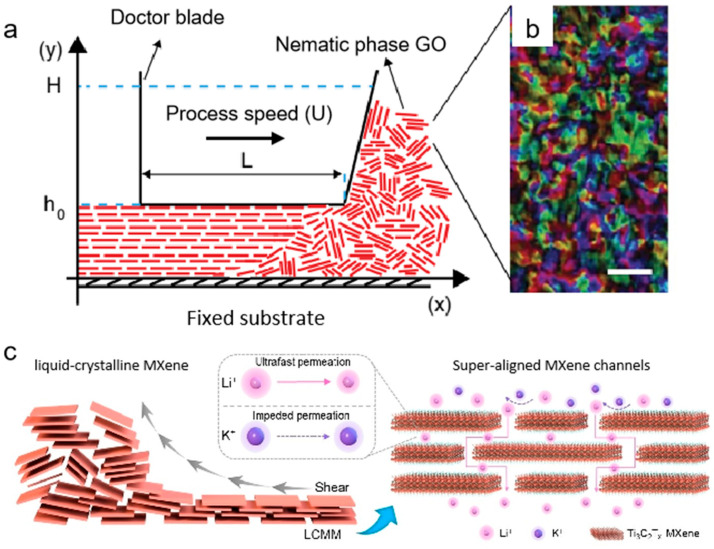
(**a**) Schematic of shear-alignment processing of nematic GO to a film: L is the width of the blade, h_0_ is the height of the channel, H is the height of the fluid in front of the blade, and U is the processing speed. (**b**) Polarized light images of fully nematic GO at 40 mg mL^−1^ (scale bar, 1 mm). (**c**) Schematic illustration of the fabrication of a liquid-crystalline MXene membrane by shearing liquid-crystalline MXene nanosheets and the corresponding separation process [69,70].

External fields, such as shear forces and electric/magnetic fields, can greatly influence the assembly of 2D material films. According to the Onsager theory, 2D nanosheet dispersions can form a nematic liquid crystal state under specific critical conditions, such as large nanosheet diameters or high dispersion concentrations [66]. As a transition phase between crystal and fluid, this liquid crystal state is characterized by long-range ordering of nanosheets in a spatial orientation, while maintaining the macroscopic flow properties of the fluid, and is able to respond to external fields, such as shear field, to produce orientation reconfiguration [67]. In the case of concentrated GO dispersions, for example, under the action of an external shear field, the nanosheets can be highly oriented along the shear direction, and the interlayer van der Waals interactions are significantly enhanced due to the decrease in the spacing of the layers and eventually form a dense stacked film with a single-crystal-like structure (Figure 7a,b) [68,69]. GO films prepared by Mainak Majumder’s group through the shear-induced assembly technique showed precise arrangement of the nano-channels [70], which greatly improve the water permeability over traditional commercial nanofiltration membranes while maintaining the efficient retention of small organic molecules. It is worth noting that this shear-field-induced orientation assembly mechanism can be also used to fabricate high-oriented MXene films. For example, the MXene membrane developed by Wang’s group achieved ultra-high selective separation of monovalent ions through the shear-induced formation of ultra-ordered nano-channel arrays, which is a breakthrough in the technological bottleneck of the traditional ion-screening membranes (Figure 7c) [71].

## 4. Applications of 2D Nanomaterials in Active Packaging

Active packaging systems are designed to interact with the environment to extend the shelf life of products, enhance food safety, or provide other functional benefits. Based on the functionality and utility of active packaging, barrier, mechanical, and antimicrobial properties are key characteristics of interest to the industry. Barrier properties are an essential core feature, as they effectively block the penetration of oxygen, carbon dioxide, water vapor, and other gases, thus slowing down the process of oxidation and deterioration of food, significantly extending the freshness of the product and maintaining its quality. On this basis, the mechanical properties of the material are an important indicator of the strength and toughness of the packaging material to ensure that the transportation and storage processes can resist a variety of external forces to maintain the integrity and reliability of the packaging. At the same time, antimicrobial properties further enhance product safety and freshness by inhibiting the growth of bacteria. The optimization and balance of these properties is essential to ensure the quality and safety of food and other products and ultimately to achieve the goal of extended shelf life.

Having established how colloidal interactions affect the assembly of 2D materials, it is now essential to examine how these interactions translate into practical applications in the field of active packaging. The unique properties of 2D materials, when properly assembled, can greatly enhance these functionalities, offering improved barrier properties, mechanical properties, and antimicrobial effects. In this section, we explore the diverse applications of 2D materials in active packaging, highlighting how their colloidal interactions enable these advanced functionalities (Table 1).

### 4.1. Enhancing Barrier Properties

**Figure 8:**
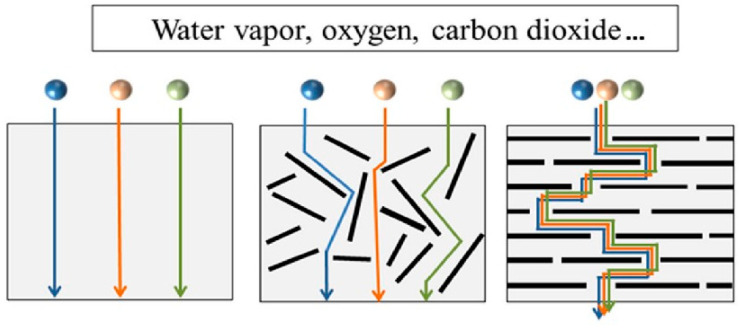
Gas molecule diffusion in packaging membranes [30].

One of the most significant advantages of 2D materials is their high aspect ratio and large surface area, which facilitate effective dispersion within polymer matrices. When integrated into polymer films, nanosized fillers establish a convoluted pathway for the diffusion of gases and moisture, thanks to the impermeable inorganic crystals present in the fillers (Figure 8). An increase in the stacking degree of the plate-like nanoparticles enhances barrier effectiveness by creating a more convoluted pathway within the polymer matrix. The colloidal interactions between the 2D materials and the polymer matrix contribute to a strong interface. Colloidal stability is essential for the successful integration of these materials. Stable dispersions ensure uniform distribution throughout the matrix, which is critical for consistent barrier performance.

**Figure 9:**
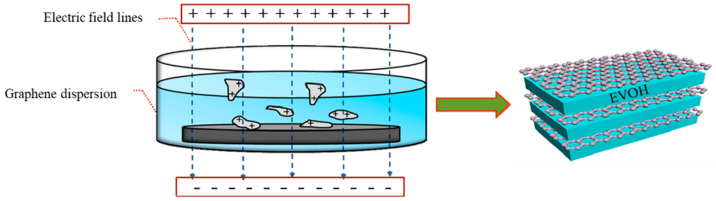
LbL assembly MTAC-rGO/EVOH film under parallel electric field [48].

The surface chemistry of 2D materials is tailored to enhance their compatibility with different polymer matrices [78,79]. Functionalizing 2D materials can result in better adhesion with the polymer. This optimization not only enhances mechanical properties but also improves barrier performance, enabling the packaging materials to more effectively safeguard their contents against moisture and gases [6]. Graphene and its derivatives are already used in packaging materials. Wang et al. employed a layer-by-layer assembly technique to create multilayer films composed of reduced graphene oxide and poly(ethylene-co-vinyl alcohol) (EVOH), as shown in Figure 9 [72]. The functionalization of [2-(methacryloyloxy) ethyl] trimethylammonium chloride (MTAC) on a rGO surface yielded positively charged MTAC-rGO nanomaterials, after which positively charged MTAC-rGO was realized on EVOH substrate under a parallel electric field. The addition of rGO extended the convoluted pathways that water molecules must traverse, which in turn provided the composite film with excellent moisture barrier properties (0.019 g m^−2^ s^−1^ atm^−1^ at a relative humidity of 99%) as well as oxygen barrier properties.

**Figure 10:**
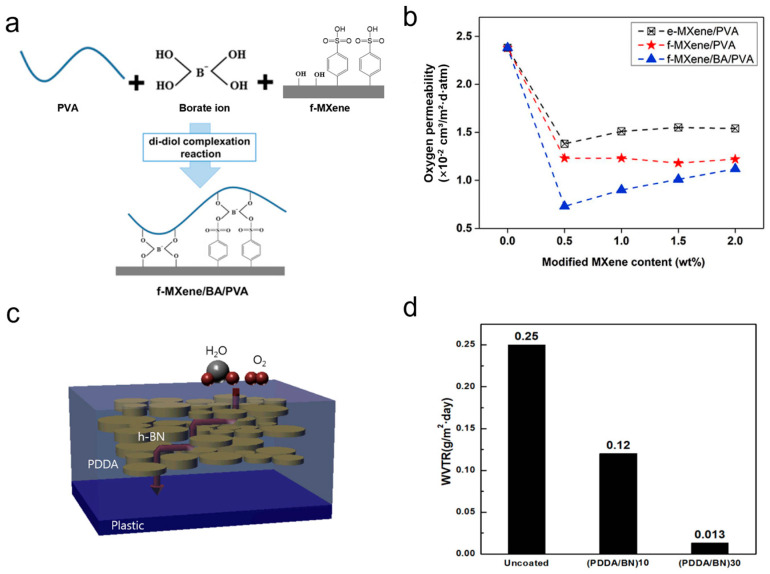
(**a**) The composite film containing low loadings of MXenes. (**b**) Oxygen permeability coefficient of composite films. (**c**) Schematic representation of diffusion path that gas molecules must pass through in the PDDA/BN gas barrier. (**d**) Water vapor transmission rate of PDDA/BN assemblies on plastic films.

The barrier properties of polymer films can be significantly enhanced through cross-linking with 2D material fillers (Figure 10a) [80]. The composite film containing low loadings of MXenes and boric acid (both at 0.5 wt. %) exhibits an oxygen permeability of approximately 0.73 × 10^−2^ cm^3^ m^−2^ d^−1^ atm^−1^, representing a reduction of about 69% in gas permeability (Figure 10b). Additionally, this film demonstrates an increase of approximately 67% in tensile strength and around 49% in modulus compared to that of a pure PVA film. These substantial improvements in gas permeability, tensile strength, and modulus indicate that this composite film holds promise as an effective packaging barrier material.

In addition, other 2D materials, such as h-BN and MoS_2_, offer new options for improving the barrier properties of composite films [81]. Electrostatic coupling of hydroxylated h-BN with a cationic polymer of polydiallyldimethylammonium chloride (PDDA) forms hydroxyl-functionalized h-BN/PDDA nanocomposites (Figure 10c). The well-dispersed and highly ordered h-BN nanosheets can reduce the water vapor transmission rate to 1.3 × 10^−2^ g m^−2^ d^−1^ (Figure 10d). The hydroxylated MoS_2_ was uniformly incorporated into polyvinyl alcohol. The helium permeability of the obtained films decreased from 169 × 10^−17^ mol m^−1^ s^−1^ Pa^−1^ to 1.8 × 10^−17^ mol m^−1^ s^−1^ Pa^−1^, indicating that the addition of hydroxyl functional group MoS_2_ significantly improved the gas resistance of the films.

### 4.2. Enhancing Mechanical Properties

Due to its exceptional mechanical properties, including high strength and rigidity, the incorporation of 2D nanomaterials can greatly enhance the mechanical performance of packaging materials. Li et al. grafted cellulose nanocrystals (CNCs) onto GO surfaces through chemical means and used this hybrid material to prepare poly(3-hydroxybutyrate-co-3-hydroxyvalerate) (PHBV) composite films. The incorporation of 1 wt.% covalently bonded CNC-GO into the PHBV matrix resulted in a ternary composite material that exhibited significant mechanical property enhancements, with the tensile strength increasing by 170.2% and the elongation at break improving by 52.1% [73]. Cai et al. (2018) developed multifunctional nanocomposite films by integrating OH-BNNSs with polyvinyl alcohol. The hydroxylation of BNNSs enhanced the van der Waals bonding between the nanosheets and the polyvinyl alcohol matrix. As a result, the addition of 0.2 wt. % OH-BNNSs led to significant improvements in the mechanical properties of polyvinyl alcohol, including a 144% increase in the Young’s modulus, a 73% increase in tensile strength, and a 109% increase in elongation at break [25].

### 4.3. Enhancing Antimicrobial Properties

Microbes are essential in the production of various foods, but they can also lead to spoilage. Foodborne pathogens such as Salmonella, Campylobacter, Staphylococcus aureus, Vibrio parahaemolyticus, Bacillus cereus, Listeria monocytogenes, and pathogenic Escherichia coli pose significant risks to human health, potentially causing illnesses like acute enteritis and diarrhea, and in severe cases, death. To mitigate these risks within the food supply chain, using antibacterial materials for packaging can effectively prolong shelf life and minimize the danger posed by these harmful microorganisms. Many 2D materials, due to their distinct physicochemical properties, are capable of inactivating microbes by penetrating bacterial membranes, disrupting their structure, leading to cytoplasmic leakage and triggering reactive oxygen species production and metabolic disturbances [82]. As a result, numerous 2D materials have been utilized to develop antimicrobial agents, including food packaging materials with enhanced antimicrobial properties. It has particularly been found that the colloidal and interfacial properties of 2D nanomaterials, including surface charge, hydrophilicity, and functional group modifications, directly influence their interaction with microorganisms and antimicrobial effectiveness, making them crucial factors in optimizing antimicrobial packaging materials.

Graphene and its derivatives have exhibited promising antibacterial properties [83]. The cytotoxic effects of graphene and GO on bacteria are attributed to physical damage resulting from direct interactions between the nanostructures and the bacterial cell membrane. However, this cytotoxicity is significantly reduced when the graphene nanosheets are coated with proteins, such as serum proteins [84]. Furthermore, graphene can be utilized on its own or combined with other functional materials to create bactericidal films or coatings [85,86]. For example, neat hyperbranched polyester films showed no antimicrobial activity while hyperbranched polyester films were incorporated with GO inhibition zones against *S. aureus* [87]. In addition, adding rGO to polyvinyl alcohol can effectively inhibit *E. coli* and *S. aureus*, respectively [88,89]. And the antimicrobial activities could be further boosted by adding silver [88].

In addition to graphene, many studies have investigated other 2D materials and their potential applications in antibacterial packaging. Zhao et al. created a food packaging material that is both cost-effective and environmentally friendly by using g-C_3_N_4_/ZnO/cellulose composites, which showed remarkable antibacterial activity. The inhibition zones measured 15.33 mm for *S. aureus.* and 14.33 mm for *E. coli* [76]. Additionally, layered double hydroxides (LDHs) that contain metal ions with bactericidal properties are frequently considered effective antibacterial materials. Cheng et al. prepared a food packaging film by dispersing ZnAl-PHBA-LDH, with a Zn:Al ratio of 4:1, into a gelatin solution. The results from antimicrobial testing indicated that the ZnAl-PHBA-LDH-gelatin film effectively inhibited the growth of *S. aureus* and *C. albicans* [90].

## 5. Conclusions

We review the advancements and challenges in the application of 2D nanomaterials for sustainable, high-performance polymer-based packaging materials. The exceptional properties of 2D nanomaterials, including their high aspect ratio, mechanical strength, and barrier capabilities, have shown significant potential in addressing critical requirements for modern packaging, such as improved barrier performance, mechanical strength, and antimicrobial properties. Key challenges, such as achieving uniform dispersion, the modulation of assembly structure, and ensuring compatibility with the polymer matrix have been discussed in detail. Recent studies highlight how surface functionalization and colloidal science-based strategies are instrumental in overcoming these barriers, paving the way for more efficient and reproducible integration processes. We conclude that 2D nanomaterials not only enhance the functionality of polymer-based packaging but also align with global sustainability goals. Future research should focus on optimizing manufacturing techniques, exploring the synergistic effects of hybrid materials, and ensuring cost-effective scalability to facilitate widespread adoption. By leveraging interdisciplinary approaches, the development of next-generation 2D material packaging can significantly reduce environmental footprints while maintaining superior performance.

## Figures and Tables

**Table 1:** Some examples of 2D materials in improving polymers’ physiochemical properties for active packaging.

Components	Barrier Property	Mechanical Property	Antimicrobial Activity	Ref.
MTAC-rGO/EVOH	OTR decreased four-fold; WVTR decreased twelve-fold	YM improved > 190%; TS improved > 30%	13.6 ± 0.5% *E. coli* survived 5.1 ± 0.2% *S. aureus* survived	[72]
PVA-2% GO films	OP and WVTR decreased by 76% and 21%	TS and EM increased by 49% and 144%	-	[16]
PHBV-CNC/GO	WVP decreased by 72.6%	TS increased by 170.2%; EAB increased by 52.1%	Inhibition zones: 3.49 mm toward *E. coli*; 3.32 mm toward *S. aureus*	[73]
PLA/GO/ZnO	UV–vis transmittance decreased > 50%	TS increased by 14.2%	Microbial inactivation: 99.2 ± 0.5% toward *E. coli*; 97.6 ± 0.8% toward *S. aureus*	[74]
MoS_2_/poly(vinyl alcohol)	Helium permeability reduced by 95%	EAB and toughness increased by 140% and 130%	-	[75]
g-C_3_N_4_/ZnO/cellulose	-	-	Inhibition zones of CNZCel: 0.45 were 176%, 191%, and 136% larger than those of ZnO, g-C_3_N_4_, and ZCel	[76]
Ti_3_C_2_ MXene/polyurethane nanocomposites	-	TS and storage modulus increased by 47.1% and 39.8%	-	[77]

Notes. YM: Young’s modulus; TS: tensile strength; EAB: elongation at break; EM: elastic modulus; WVP: water vapor permeability; WVTR: water vapor transmission rate; OP: oxygen permeability; OTR: oxygen transmission rate.
